# Addition of bevacizumab to first-line chemotherapy in advanced colorectal cancer: a systematic review and meta-analysis, with emphasis on chemotherapy subgroups

**DOI:** 10.1186/1471-2407-12-89

**Published:** 2012-03-13

**Authors:** Ligia Traldi Macedo, Andre Bacellar da Costa Lima, Andre Deeke Sasse

**Affiliations:** 1 Centre for Evidences in Oncology (CEVON) - Clinical Oncology Division, State University of Campinas (UNICAMP), Vital Brasil 251, 13083888 Campinas, Brazil

## Abstract

**Background:**

Bevacizumab has an important role in first-line treatment of metastatic colorectal cancer. However, clinical trials studying its effect have involved distinct chemotherapy regimens with divergent results. The aim of this meta-analysis is to gather current data and evaluate not only the efficacy of bevacizumab, but also the impact of divergent backbone regimens.

**Methods:**

A wide search of randomized clinical trials using bevacizumab in first-line metastatic colorectal cancer was performed in Embase, MEDLINE, LILACS and Cochrane databases. Meeting presentations and abstracts were also investigated. The resulting data were examined and included in the meta-analysis according to the type of regimen.

**Results:**

Six trials, totaling 3060 patients, were analyzed. There was an advantage to using bevacizumab for overall survival (OS) and progression-free survival (PFS) (HR = 0.84; CI: 0.77-0.91; P < 0.00001 and HR = 0.72; CI: 0.66-0.78; P < 0.00001, respectively). However, heterogeneity of results was very high for both outcomes, and subgroup analyses supported the OS advantage with bevacizumab restricted to irinotecan-based regimens. Infusional fluorouracil subsets involved a minor proportion, and did not demonstrate statistical benefit in PFS or OS. Regarding toxicity, higher rates of grades 3-4 hypertension, bleeding, thromboembolic events and proteinuria were uniformly observed with bevacizumab, leading to increased treatment interruptions (HR = 1.47; P = 0.0004).

**Conclusions:**

Bevacizumab has efficacy in first-line treatment of advanced colorectal cancer, but the current data are insufficient to support efficacy in all regimens, especially infusional fluorouracil regimens, like FOLFIRI and FOLFOX.

## Background

Colorectal cancer is currently the third most diagnosed cancer in men and the second in women worldwide, with an estimate of over 1.2 million new cases and 608,700 deaths in 2008 [1]. In the attempt of disease control, target therapy has been a matter of extensive research. Anti-angiogenesis is one of the pivotal theories involved in this approach, ever since the discovery of increased vascularity as a probable key for tumour progression [2,3]. One of the main pathways associated with the anti-angiogenic process is the vascular endothelial growth factor (VEGF) family, with high expression of its receptors observed not only in colorectal neoplasms, but in a wide variety of distinct tumours [4]. This fact led to the development of many VEGF inhibitors, amongst which bevacizumab is one of the most common.

Bevacizumab in colorectal cancer was studied initially in the metastatic setting, and was approved by US Food And Drug Administration (FDA) in 2004, based on a survival benefit noted in the AVF2107 trial [5] with the Saltz' irinotecan, 5-fluorouracil and leucovorin (IFL) regimen [6]. However, a similar benefit was not seen in the recent single-centre randomised trial by Stathopoulos et al, analysing bevacizumab with irinotecan and bolus fluorouracil [7].

Other recent trials have also failed to demonstrate the same statistically significant results in survival, particularly with other backbone regimens, such as isolated capecitabine or oxaliplatin-containing regimens. One of the most mentioned studies regarding oxaliplatin-based chemotherapy is a prospective, double-blind randomised trial of 1400 patients evaluating bevacizumab and the FOLFOX or XELOX regimen in first-line treatment [8]. The results of this study confirmed a significant relative benefit of 17% for disease-free survival, but overall survival (OS) did not achieve statistical significance. Currently, the benefit on OS with the use of oxaliplatin is limited to the second-line setting, applying higher doses of bevacizumab [9].

Therefore, the use of bevacizumab in colorectal metastatic disease has been a topic of much debate. All studies available so far, when analysed individually, were unable to reach the same conclusion. Thus, cost-effectiveness is also unclear. This has led to distinct practice guidelines from country-to-country, according to reimbursement policies [10]. Probable causes for such conflict could be the unavailability of an optimal, standard therapy for this disease, to which a comparison of bevacizumab would facilitate more accurate data [11]. Moreover, the introduction in clinical practice of cetuximab [12-14] and panitumumab [15], monoclonal antibodies against epidermal growth factor receptor (EGFR), raised more questions concerning which target agent should be preferred in the first-line approach.

With the advent of new randomised trials, the objective of this meta-analysis is to gather current data and evaluate the effect of bevacizumab in first-line therapy, focusing on each backbone regimen.

## Methods

### Search Strategy

Articles published or presented from August 2002 to March 2011 were identified by a thorough investigation of electronic databases including PubMed/MEDLINE, EMBASE, LILACS, and The Cochrane Library. Meeting websites from ASCO, ESMO, and the World Congress on Gastrointestinal Cancer from 2005 to 2011 were also examined. A sensitive search strategy was performed through terms related to *colorectal neoplasms, bevacizumab*, and *randomised trials *in all fields. There were no limits established for language, methodological characteristics or year of publication. Lists of references from relevant articles were scanned and all additional studies of potential interest were retrieved for further analysis.

### Selection Criteria

The goal of this study was to identify all published randomised, controlled clinical trials with a corresponding design of comparison to chemotherapy with or without bevacizumab for metastatic colorectal cancer, in previously untreated patients. To avoid bias related to drug interactions, studies involving the use of other targeted agents were excluded.

Two reviewers (LTM, ADS) examined the list of references and individually selected the studies. A meeting was then held to achieve consensus.

### Data Extraction

For distinguishing purposes, the name of the first author and the year of publication of the article were indicated. Two reviewers (LTM, ABL) independently extracted the data from all selected trials. A third reviewer (ADS) was consulted in case of divergence.

OS and progression-free survival (PFS) were the main outcomes analyzed. Other points of interest were objective response rate (ORR) and toxicity, measured by the Common Toxicity Criteria (CTC) scale and retrieved as published.

The preferred method of data extraction was to directly collect the original hazard ratios (HR) described in each study. If data reported were insufficient, the corresponding authors were contacted with a request for the pending information. Alternatively, data could be estimated indirectly applying either the reported number of events and the corresponding P-value for the log-rank statistics, or by transcription of survival curves as suggested by Parmar and colleagues [16]. In this case, calculations were made through a spreadsheet provided by Tierney and colleagues [17]. The original survival curves from electronic publications were enlarged so that reading errors could be prevented, and data extraction was based on reading off electronic coordinates for each variable.

### Statistical Analysis and Synthesis

Two reviewers (LTM, ABL) extracted details regarding methodological dimensions empirically linked to bias as described by Deeks and colleagues [18]. Special focus was given to the availability of information about the sequence of randomisation, blinding and existence of placebo, the intention-to-treat analysis (ITT), and the source of funding. According to the resulting data, the methodological quality of each trial was validated. These details were then applied in subgroups, and sensitivity analyses were performed to test the stability of conclusions.

Meta-analyses for this study were conducted with RevMan 5.0 software (Cochrane Collaboration's Information Management System). Analyses of data consisted of HR for time-to-event outcomes comparisons, and odds ratio (OR) for dichotomous variables for which 95% confidence intervals (CI) were calculated and presented in forest plots. The diamond at the bottom of the plot summarizes the best estimate of pooled valid outcomes (the width representing its corresponding 95% CI). The effect of the treatment for each single study was expressed as a ratio of the bevacizumab-containing arm over the chemotherapy alone arm. A HR value of less than one and an OR value of more than one meant a benefit for the association of bevacizumab.

Statistical heterogeneity was evaluated through chi-square test [19], and expressed in I*^2 ^*index, as described by Higgins and colleagues [20]. If heterogeneity was detected (I*^2 ^*> 35%), a possible explanation for it was investigated. An individual analysis was performed in the presence of a detectable cause, otherwise data were preferably not pooled. Publication bias was evaluated according to Egger's test [18].

## Results and Discussion

### Search Results

**Figure 1:**
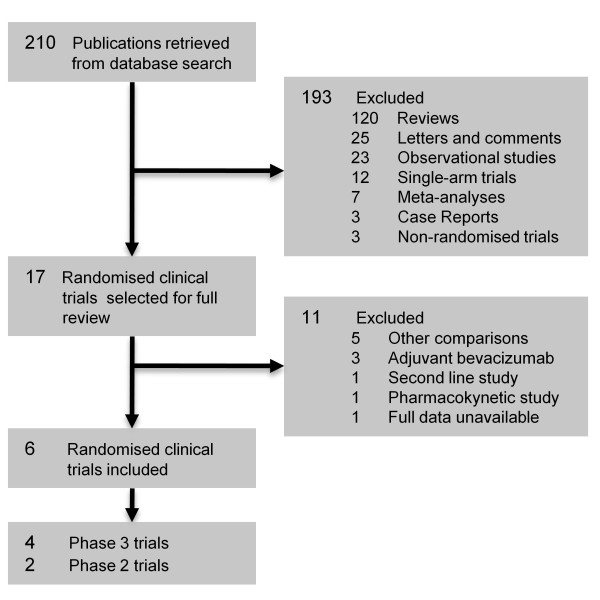
**Selection of included trials**.

The literature search, involving articles published from August 2002 to March 2011, generated 7 potential trials [21-27]. One study was not included since it was written in Chinese, and its full text was not available in press [27]. The selection procedure is further summarized in Figure 1. A total of two phase II and four phase III trials were incorporated, comprising 3060 enrolled patients. Table 1 describes the main details of the selected studies.

**Table 1:** Trial Characteristics

	*Irinotecan-based*		*Oxaliplati n-based*	*Fluorouracil alone*		
Study	**Hurwitz, 2004 (AVF2107) **[26]	**Stathopoulos, 2010 **[24]	**Saltz, 2008 (NO16966) **[25]	**Kabinnavar, 2003 **[21]	**Kabinnavar, 2005 **[22]	**Tebutt, 2010 (MAX) **[23]
**No. of patients (placebo/bev)**	411/402	108/114	701/699	36/68	105/104	156/156
**Phase**	III	III	III	II	II	III
**Randomization**	Adequate	Adequate	Adequate	Adequate	Adequate	Unclear
**Study Population**	First-line	First-line	First-line	First-line	First-line, elderly	First-line, elderly
**ITT Analysis**	Yes	No	Yes	No	Yes	Yes
**Blinding**	Yes	No	Yes	No	Yes	Yes
**Multicentric**	Yes	No	Yes	Yes	Yes	Yes
**Alpha error**	Yes	Yes	Yes	NS	Yes	Yes
**Beta error**	Yes	Yes	Yes	NS	Yes	Yes
**Withdrawals**	NS	Described	Described	Described	Described	Described
**Regimens**	Saltz IFL	Irino 135 mg/m2 + 5 FU 500 mg/m2 + LV 200 mg/m2 every 21 d	XELOX or FOLFOX4	Roswell- Park	Roswell- Park	Capecitabine
**Bev schedule**	5 mg/kg every 14 d	7.5 mg/kg every 21 d	7.5 mg/kg every 21 d (XELOX) or 5 mg/kg every 14 d (FOLFOX4)	10 mg/kg every 14 d (32 patients) 5 mg/kg every 14 d (33 patients)	5 mg/kg every 14 d	7.5 mg/kg every 21 d
**Maintenance**	Until Progression	Until Progression	Until Progression	48 Weeks	Until Progression	Until Progression
**Sponsor**	Industry	NS	Industry	NS	Public	Public/Industry

Description of the main trials' characteristics and details prompt to bias.

Abbreviations: 5FU: fluorouracil; bev: Bevacizumab; d: days; Irino: Irinotecan; ITT: Intention to Treat; LV: leucovorin; NS: not stated.

### Progression-free Survival

From the six trials selected, five included the related data, totalling 2938 patients. The association of bevacizumab demonstrated uniform benefit (HR = 0.72; 95% CI: 0.66-0.78; P < 0.00001) although high heterogeneity between trials was observed (*I² *= 69%; P = 0.01).

For better interpretation of this discrepancy, subgroup analyses were performed according to the chemotherapy regimen studied.

**Figure 2:**
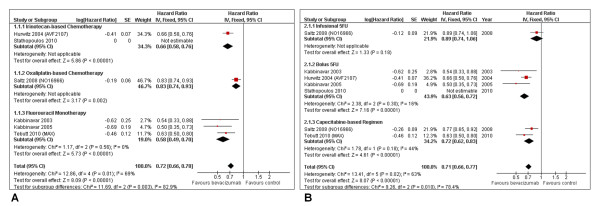
**Progression-free survival**. Progression-free survival in subgroup analyses per type of cytotoxic agent associated (A) and type of fluoropyrimidine administration (B).

The first comparison, sorted by cytotoxic agents (Figure 2A), demonstrated an advantage of bevacizumab with either 5-FU monotherapy or irinotecan-based chemotherapy, while oxaliplatin -based treatment, though beneficial, was less intense. The difference between subgroups was further confirmed by the statistical significant heterogeneity (I² = 82.9%; P = 0.003).

A second comparison involved results by type of fluoropyrimidine administration (Figure 2B), in which the NO16966 study was divided into its XELOX and FOLFOX subsets. In this case, PFS was significantly favorable for bolus 5-FU studies (HR = 0.63; 95% CI: 0.56-0.72; P < 0.00001; heterogeneity: *I² *= 16%; P = 0.30) as well as capecitabine regimens (HR = 0.72; 95% CI: 0.62-0.83; P < 0.00001; heterogeneity: *I² *= 44%; P = 0.18). Infusional 5-FU, however, in the single subgroup analysis of FOLFOX, did not present statistical advantage (HR = 0.89; 95% 95% CI: 0.74-1.06; P = 0.18).

### Overall Survival

With regards to OS, only one study [21] did not access this outcome and was excluded. The results indicated an advantage to the use of bevacizumab (HR = 0.84; 95% CI: 0.77-0.91; P < 0.00001), though heterogeneity was also observed (*I² *= 60%; P = 0.04).

**Figure 3:**
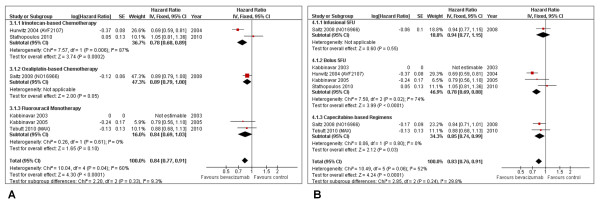
**Overall survival**. Overall survival in subgroup analyses per type of cytotoxic agent associated (A) and type of fluoropyrimidine administration (B).

In the cytotoxic agent subgroup evaluation (Figure 3A), despite conflicting results between both trials involved (*I² *= 87%; P = 0.006), the increment in OS occurred only for irinotecan-based regimens (HR = 0.78; 95% CI: 0.68-0.89; P = 0.0002), owing to the influence of one single study [26]. Neither 5-FU monotherapy, nor oxaliplatin-based treatments presented statistically significant data.

Regarding the type of fluoropyrimidine administration (Figure 3B), bolus 5-FU (HR = 0.78; 95% CI: 0.69-0.88; P < 0.00001) and capecitabine schemes (HR = 0.85; 95% CI: 0.74-0.99; P = 0.03) were beneficial. The single subset of infusional 5-FU did not reach significance.

### Overall Response Rate

**Figure 4:**
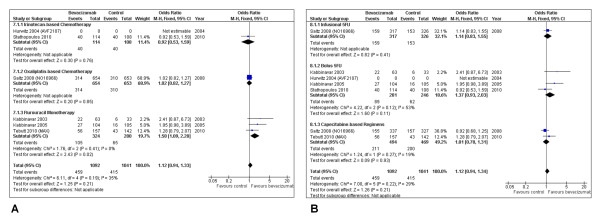
**Overall response rate**. Overall response rate in subgroup analyses per type of cytotoxic agent associated (A) and type of fluoropyrimidine administration (B).

In contrast to the previous outcomes, the meta-analysis of data involving ORR did not demonstrate favourable overall results (OR = 1.12; 95% CI: 0.94-1.33; P = 0.21). Fluorouracil monotherapy, on the other hand, was the only subset with statistical significance, demonstrating a higher ORR with the addition of bevacizumab (OR = 1.58; P = 0.02). All subgroup rates are further described in Figure 4A and 4B.

### Treatment-related Toxicity

**Figure 5:**
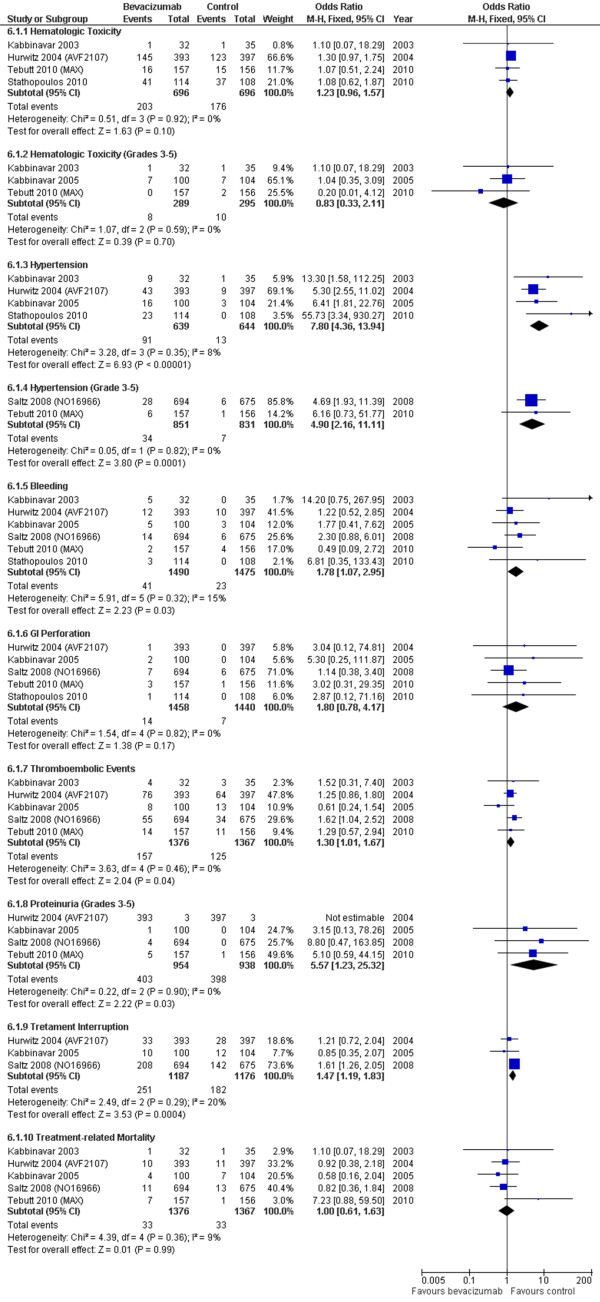
**Meta-analysis of toxicity**.

In contrast to PFS or OS, results regarding toxicity were not heterogeneous, with I^2 ^indexes ranging from 0% to 20% (Figure 5). The addition of bevacizumab was linked to increase rates of hypertension, proteinuria, bleeding and thromboembolic events, also leading to a slight increment on treatment interruptions (HR = 1.47; 95% CI: 1.19-1.83; P = 0.0004), particularly higher in the oxaliplatin-containing study [25]. Other variables, such as hematologic toxicity and gastrointestinal perforation, were not statistically significant.

Treatment-related mortality, in this specific study for first-line therapy, was not significantly influenced by the addition of bevacizumab.

## Conclusions

Several randomised trials have tested bevacizumab in diverse combinations of oxaliplatin, irinotecan, 5-FU or capecitabine. By analysing these data, it is possible to observe high heterogeneity amongst studies. There are two hypotheses to explain these differences: firstly, the fact that trials being as divergent in their results as in their designs (number of patients, selection criteria, bevacizumab dose) could influence the data and consequently introduce bias. The second explanation is a possible interaction of cytotoxic agents with VEGF-inhibitors like bevacizumab.

Evidence to support this second hypothesis is evident in the subgroup analyses of this study. Irinotecan based-regimens appear to be the most advantageous with regards to OS. The first study to evaluate this combination, AVF2107 [5], enrolled 813 patients in a double-blind, randomised phase III study involving IFL regimen, and revealed a clear advantage on OS (4.7 months) as well as PFS with the addition of bevacizumab at 5 mg/kg. However, other subsequent study failed to demonstrate the same response pattern. The recent single-centre randomised trial by Stathopoulos et al [7] with 222 patients, compared the combination of bevacizumab at 7.5 mg/kg to a bolus regimen of irinotecan and fluorouracil, both every 21 days. Though the limitations of this trial should not be taken aside (single-centre, unblinded, and a particular irinotecan-based regimen), OS and ORR were not advantageous in the regimen. It is also important to consider that current studies indicate infusional 5-FU regimens like FOLFIRI to be more beneficial than IFL. The protocol amendment from BICC-C, a trial originally planned to compare distinct irinotecan-based therapies, randomised 117 patients to FOLFIRI plus bevacizumab, 5 mg/kg every 2 weeks, or IFL plus bevacizumab, 7.5 mg/kg every 3 weeks [28]. Updated analyses described a significant superiority on OS (28 months v 19.8 months; P = 0.037) for the infusional treatment, versus bolus regimen, at the cost of increased grade 3/4 hypertension (12.5% v 1.7%). Whether the presence of bevacizumab is an advantage to this combination and what should be the extent of such benefit are still uncertain issues.

Regarding oxaliplatin-based therapies, results are even more contradictory. In the first-line setting, there is only increased PFS supported by the single-study NO16966 [8], in the XELOX subgroup. The same marginal benefit in PFS without impact in OS from oxaliplatin association seems to extend to other trials involving VEGF-inhibitors. Two recent studies associating PTK/ZK, an oral antibody, to FOLFOX in first- [29] and second-line therapies [30], and one trial with the addition of the multikinase inhibitor cediranib to FOLFOX or XELOX [31], presented a similar pattern of results. This evidence could reinforce the impression that oxaliplatin might not be an ideal partner for such target inhibitors. Nonetheless, there are studies indicating otherwise. The E3200 trial [9] evaluated the addition of bevacizumab, with a higher dose of 10 mg/kg, to the FOLFOX4 scheme. Amongst 829 patients enrolled for second-line treatment, this study demonstrated statistically significant gain on OS (10.8 v 12.9 months; P = 0.0011) as well as ORR (8.6% v 22.7%). Thus, data available are insufficient to reach a conclusion about whether the addition of bevacizumab to an oxaliplatin-based regimen, especially with FOLFOX, could be beneficial in chemotherapy-naive patients.

Furthermore, when combined only with 5-FU, bevacizumab demonstrated the highest benefit in PFS, despite the absence of significant results for OS, in the subgroup meta-analysis previously presented. In contrast to the other agent subsets, no heterogeneity was observed. This effect over PFS without impact on survival could be explained by the selected population, restricted to the elderly, usually unfit for other combination therapies.

The addition of bevacizumab to palliative chemotherapy in advanced colorectal cancer is a paradigm left unsolved. The overall benefit of such combination was already attested in three global meta-analyses previously published. The two first major systematic reviews assessed five trials either in first- or second-line settings [32,33]. A comparison performed by Welch and colleagues in 2010 [33] demonstrated significant reduction in mortality (HR = 0.79; 95% CI: 0.69-0.90; P = 0.0005), as well as progression (HR = 0.63; 95% CI: 0.49-0.81; P = 0.0004), reass!uring the former findings from the first published meta-analysis, by Cao and colleagues [32]. With the advent of one new randomised trial, a subsequent global review was repeated by Galfrascoli et al [34], maintaining global benefit observed in OS and PFS.

The delicate point of discussion from all those former systematic reviews is that this advantage is not uniform. Individual results are contradictory when assessed in particular, since the regimens of comparison are distinct. Most trials have failed to prove statistical benefit for survival, in light of the incremental cost and toxicity. Considering that those differences should not be underestimated, the main focus of this current meta-analysis was the separate comparison of backbone chemotherapies, according to cytotoxic elements involved and pattern of 5-FU administration.

Although the present study did not show higher fatal adverse events, a recent meta-analysis involving 16 clinical trials with bevacizumab for solid tumours described a significant increase in treatment-related mortality rate (2.5% v 1.7%; P = 0.01), particularly in association to taxanes and platinum agents (RR = 3.49; 95% CI: 1.82-6.66; incidence, 3.3% vs 1.0%) [35].

These divergent results led to distinctly different practices throughout health systems: whereas bevacizumab is approved for management of metastatic colorectal neoplasm by the FDA and European Medicines Agency (EMA), guidance recently published from the United Kingdom's National Institute for Health and Clinical Excellence (NICE) according to data and cost analyses, indicates a refusal to approve the use of such medication.

All studies currently available, including the overall results of this meta-analysis, lead to the conclusion that bevacizumab is an effective agent for first-line treatment for metastatic colorectal cancer. Nevertheless, its effectiveness is observed in limited subsets as bolus fluorouracil, capecitabine-regimens, and in combination with irinotecan. Further studies involving infusional chemotherapy and oxaliplatin combinations are required to better characterize the pharmacological interference from companion agents.

## Abbreviations

5-FU: Fluorouracil; Bev: Bevacizumab; CI: Confidence interval; CTC: Common Toxicity Criteria; d: Days; EMA: European Medicines Agency; FDA: Food and Drug Administration; FOLFIRI: Infusional regimen of fluorouracil, combined with irinotecan and leucovorin FOLFOX, infusional regimen of fluorouracil: combined with oxaliplatin and leucovorin; HR: Hazard ratio; IFL: Saltz' irinotecan, 5-fluorouracil and leucovorin bolus regimen; Irino: Irinotecan; ITT: Intention-to-treat analysis; LILACS: Literature in the Health Sciences in Latin America and the Caribbean; LV: Leucovorin; MEDLINE: Medical Literature Analysis and Retrieval System Online; NICE: National Institute for Health and Clinical Excellence; NS: Not stated; OS: Overall survival; PFS: Progression-free survival; OR: Odds ratio; ORR: Objective response rate; RR: Risk ratio; VEGF: Vascular endothelial growth factor; XELOX: Capecitabine and oxaliplatin.

## Competing interests

The authors involved in the development of this manuscript have no conflicts of interest to declare, nor funding sources from third parties.

## Authors' contributions

LTM: participated in the study design, data extraction, article selection, statistical analysis and manuscript preparation; ABCL: participated in the study design, article selection and data extraction; ADS: conceived of the study, participated in the study design, data extraction, article selection, statistical analysis and coordination; All authors read and approved the final manuscript.

## Pre-publication history

The pre-publication history for this paper can be accessed here:

http://www.biomedcentral.com/1471-2407/12/89/prepub
